# Larvicidal Activity of Brusatol Isolated from *Brucea javanica* (L) Merr on *Culex quinquefasciatus*

**Published:** 2019-04

**Authors:** Dwi SUTININGSIH, Nurjazuli NURJAZULI, Djoko NUGROHO, Tri Baskoro Tunggul SATOTO

**Affiliations:** 1. Department of Epidemiology and Tropical Disease, Faculty of Public Health, Diponegoro University, Semarang, Indonesia; 2. Department of Environmental Health, Faculty of Public Health, Diponegoro University, Semarang, Indonesia; 3. Department of Biostatistics, Faculty of Public Health, Diponegoro University, Semarang, Indonesia; 4. Department of Parasitology, Faculty of Medicine, Gadjah Mada University, Yogyakarta, Indonesia

**Keywords:** Larvicide, Brusatol, *Brucea javanica* (L) Merr, *Culex quinquefasciatus*

## Abstract

**Background:**

Vector control is still emphasized on the using of chemical insecticide which can cause death of non-target organisms, pollution and vector resistance. Therefore, natural insecticides/larvicides are an alternative to using chemical insecticides to control the mosquito vector.

**Methods:**

Brusatol was isolated from the seeds of Makassar Fruit (*Brucea javanica* L. Merr). *Culex quinquefasciatus* larvae were divided into 3 groups, i.e. 6 testing groups and one negative and positive control group. In the negative control group, the larvae were treated with 100 ml aquadest and positive control was treated with temephos 1 ppm. After 24 hours, dead larvae were calculated and the percentage of death was determined. The dead larvae were then examined for morphological changes using a light microscope.

**Results:**

The higher of the concentration level of brusatol, the higher number of the death of *Cx. quinquefasciatus* larvae (*P*<0.05). The value of brusatol Lethal Concentration 50 to larvae *Cx. quinquefasciatus was* 0.010 ± 0.122 and value of brusatol Lethal Concentration 90 to larvae *Cx. quinquefasciatus* was 0.654 ± 0.081 ppm. The higher the concentration of brusatol, the morphological damage of *Cx. quinquefasciatus* larvae was getting worse and widespread to cause damage to the digestive tract and cuticle.

**Conclusion:**

Brusatol isolated from the seed of *B. javanica* (L) Merr have larvicidal activity to the *Cx. quinquefasciatus* which is potential to be natural larvicide.

## Introduction

The efforts of vector control in order to suppress the incidence of vector-borne disease to this day are still being attempted. The use of chemical insecticides and synthetic organism widely in vector control is endangering the environment and causing he resistance in vector (1). Therefore, it is necessary to find the alternative way to control the vector disease, that is using natural insecticides/larvicides that come from plants (2). Indonesia is rich of plants that have potential as natural insecticides/larvicides, and many of them have not yet proven their effectiveness scientifically (3).

*Brucea javanica* (L) Merr is known rich for quassinoid compounds, such as brusatol, bruceantine and bruceine (4). Several researches on quassinoids showed high potential as natural source of insecticides/larvicides. Quassinoids proven as compounds that responsible to the change of feeding behavior and growth regulation on insects (5). Quassin, simalikalactone D, bruceantine, gluocarubinone and isobruceine A have been proven to be effective as antifeedant against Mexican bean beetles (*Epilachna varivestis)*, cabbage caterpillar (*Plutella xylostela*) and south caterpillar (6). Brusatol isolated from *B. javanica* (L) Merr has insecticidal effect and antifeedant on larvae instar 3 of *Spodoptera exigua* (7). Brusatol can induce apoptosis on insect cell line IOZCAS-*Spec-*II and Sf21 characterized by DNA fragmentation, kaspace-3 activation and the release of cytochrome-c enzyme from mitochondria (7).

Larvicidal activities of brusatol on *Aedes aegypti* larvae with Lethal Concentration value of 50 and 90 (LC_50_, LC_90_) are 0.669 ± 0.106 ppm and 8.331 ± 0.060 ppm (8). The treatment with various brusatol concentration on *Ae. aegypti* larvae showed neurotoxic syndromes that were exitacy, convulsion, tremor and paralysis (8). Another quassinoid compound contained in *B. javanica* (L) Merr that is bruceine A also shown to prohibit the growth and development of *Ae. aegypti* larvae into pupae or adults marked with the damage on cuticles and necrosis on gastrointestinal epithel cells and respiratory tracts (9). Bruceine A isolated from the seed of Makasar fruit (*B. javanica* L. Merr) is also neurotoxic on larvae of *Ae. aegypti* through the prohibition on acetylcholinesterase enzyme and Voltage Gated Sodium Channel/VGSC gene (10). Bruceine A and brusatol from Makasar fruit have the potential to be developed as natural larvicides and insecticides to vector disease control.

The purpose of this research was to find out the larvicidal activity of brusatol on *Cx. quinquefasciatus* and its morphological changes/damages.

## Methods

This research was conducted from May to September 2017. The extraction and isolation of brusatol from Makasar fruit was done in Pharmaceutical Biology Laboratory, Faculty of Pharmacy, Gadjah Mada University, Yogyakarta, Indonesia. The examination of larvicidal activity and morphological changes was conducted at the Parasitology Laboratory, Faculty of Medicine, Gadjah Mada University, Yogyakarta, Indonesia. Larvae *Cx. quinquefasciatus* instar 3 up to the beginning of instar 4 were obtained and reared in the Parasitology Laboratory, Faculty of Medicine, Gadjah Mada University, Yogyakarta, Indonesia.

### Extraction and isolation brusatol from B. javanica L. Merr

Brusatol extraction and isolation from the seeds of Makasar fruit was using Zhang et al. (7) method with slight modifications. Makasar fruit obtained from the traders of medicinal plants (Merapi Herbal Yogyakarta), identified in the Laboratory of Pharmaceutical Biology, Faculty of Pharmacy Gadjah Mada University, Yogyakarta, Indonesia. Chemicals and solvents used for the extraction and isolation of brusatol that are ethanol, methanol, chloroform, ethyl acetate and H_2_O were provided with the highest analytical quality obtained from Merck suppliers. *Brucea javanica* (L) Merr dried seeds (5 kg) made it into powder, shaken with EtOH-H_2_O (15 L), the solvent was evaporated in vacuum, and the extracts were combined and concentrated, followed by suspending in H_2_O. The aqueous layer was further extracted with Petroleum ether, CHCl_3_, EtOAc and n-Butanol. The CHCl_3_ layer was evaporated under vacuum to afford extracts. This extract was re-suspended with CHCl_3_, and then chromatographed on a silica gel column (400 g, 200–300 mesh) by eluting successively with CHCl_3_ containing increasing amounts of MeOH (1:0, 50:1, 20:1, 10:1, 5:1 and 0:1). The result in the organic layer was taken and evaporated to obtain a concentrate, and then made a solution of MeOH (100–250 mL) at a temperature of 60 °C and then stored at room temperature. MeOH solution above when it was allowed to stand at room temperature to form crystalline compounds that expected as brusatol. Further separation was by filtration. The rest of the filtrate / residue was separated by thin layer of chromatography (TLC). The filtrate / residue was evaporated. Further separation was performed if it was necessary.

### Larvicidal test of brusatol

The larvicidal test was carried out using the bio-assay method according to WHO (11) with slight modifications. The third instar larvae up to the beginning of instar 4 of *Cx. quinquefasciatus* were acquired and allowed to develop at the Laboratory of Parasitology, Faculty of Medicine, University of Gadjah Mada. A preliminary test was conducted to determine the range of concentrations of brusatol that could be deadly to larvae of *Cx. quinquefasciatus* instar 3 up to the beginning of in-star 4. In further tests, temephos was used as a positive control at a concentration of 1 ppm, whereas the negative control consisted of 100 mL of distilled water only. The selection of temephos dosage (1 ppm) was based on lethal damage consideration used in the field. A total of 25 larvae of *Cx. quinquefasciatus* instar 3 up to the beginning of instar 4 were used in each treatment medium and control, replicated four times. After 24 h, the dead larvae were counted. The temperature and pH of the media and humidity in the room were measured at the beginning and the end of the study.

### Larvicidal activity of brusatol

Based on the isolation method from Zhang et al. (7), 150 mg of brusatol isolated from 5 kg of Makasar fruit were obtained. The purity of the brusatol isolates analyzed by Thin Layer Chromatography (TLC) shows the presence of a purple single spot with an Rf value of 0.79 on 366 nm UV observation. The environment considered in this study was the pH of the media, media temperature, and humidity. These were measured at the beginning and end of the study as pH 7, 25 °C, and 70%–71%, respectively

### Morphology test

*Culex quinquefasciatus* larvae in instar 3 up to the beginning of instar 4 were acquired and cultured at the Laboratory of Parasitology, Faculty of Medicine, University of Gadjah Mada. Morphological tests were conducted in accordance with the method reported by Sharma et al. (12) with slight modifications. Larvae were placed in a plastic cup containing 100 ml of water and various concentrations of brusatol or 1 ppm of the positive control temephos. Negative controls were treated with distilled water. Dead larvae were collected after 24 h of treatment for the examination of morphological changes under light microscopy. Larvae were scrutinized after mounting with Hoyer’s medium and morphological changes in body segments including the head, setae, cuticle, abdomen, and anals gills were observed, photographed and compared with those of the controls.

### Data analysis

The percentage of deaths of *Cx. quinquefasciatus* larvae was expressed by Mean ± Standard Error of Mean (SEM). The value of LC_50_ and LC_90_ brusatol was determined by using Probit regression analysis with SPSS version 24 (Chicago, IL, USA). To find out the difference between concentrations of brusatol in killing the larvae of *Cx. quinquefasciatus*, we used Kruskal Wallis test followed by Mann Whitney. Statistically significant differences were indicated at *P*<0.05. The data from observation on morphological changes were descriptively analyzed (13).

## Results

The percentage of deaths of *Cx. quinquefasciatus* larvae presented in Table 1. The average values of Lethal Concentration 50 (LC_50_) and Lethal Concentration 90 (LC_90_) of brusatol against larvae of *Cx. quinquefasciatus* were 0.010 ± 0.122 ppm and 0.654 ± 0.081 ppm respectively.

**Table 1:** Percentage of the mortality of *Cx. quinquefasciatus* larvae treated with brusatol, temephos of 1 ppm and control after 24 h of observation

***No.***	***Concentration brusatol (ppm)***	***No. of larva***	***Mortality of larvae on each replicatoin***				***Mean***	***Mortality ± SEM (%)***
			***I***	***II***	***III***	***IV***		
1.	0.1	25	14	15	18	17	16.00	64 ± 0.55*
2.	0.3	25	18	20	21	20	19.75	79 ± 0.38*
3.	0.9	25	19	20	20	23	20.50	82 ± 0.52*
4.	2.7	25	23	20	22	24	22.25	89 ± 0.51*
5.	8.1	25	23	24	22	24	23.25	93 ± 0.29*
6.	24.3	25	25	25	25	25	25.00	100 ± 0.00*
7.	Temephos 1ppm	25	25	25	25	25	25.00	100 ± 0.00
8.	Control	25	0	0	0	0	0.00	0 ± 0.00

* P<0.05 with Kruskal Wallis and Mann Whitney test; ppm: part per million; SEM: Standard Error of Mean

### Morphological changes of Cx. quinquefasciatus

**Fig. 1:**
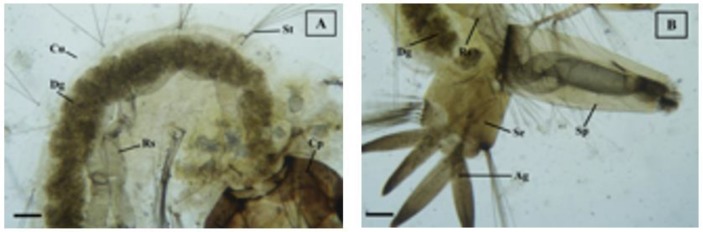
Control of *Cx. quinquefasciatus* larvae. Digestive and respiratory tract complete and well (A). Anterior body, (B). Posterior body (100x). Cp: caput, Dg: digestive tract, Rs: respiratory tract, Sp: siphon, Se: sandle, Cu: cuticle, Ag: anal gills, St: setae

**Fig. 2:**
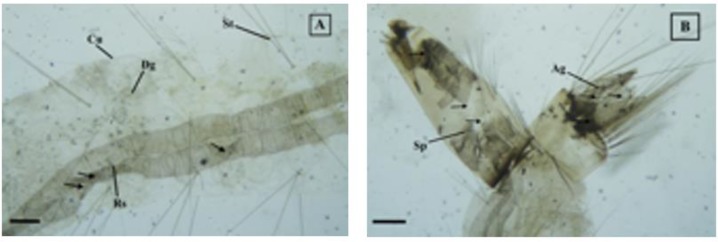
Larvae of *Cx. quinquefasciatus* treated with brusatol, (A). Brusatol concentration of 0.9 ppm. Respiratory tube folded, cuticle and feather setae detached in some parts, (B). Brusatol concentration of 0.1 ppm. Siphon and anal gills broken and detached (100x). Cp: caput, Dg: digestive tract, Rs: respiratory tract, Sp: siphon, Se: sandle, Cu: cuticle, Ag: anal gills, St: setae

The morphological observation of *Cx. quinquefasciatus* larvae after complete brusatol and control treatment is presented in Table 2, Figs. 1 and 2.

**Table 2:** Morphological changes of *Cx. quinquefasciatus* larvae with control and brusatol treatment at various concentrations

***Treatment***	***Morphological changes in Cx. quinquefasciatus larvae***
Brusatol 24.3 ppm	Damages on cuticle, digestive and respiratory tracts especially in the siphon.
Brusatol 8.1 ppm	The body of the larvae and the head were blackened, the digestive tract was damaged by a black spot, the respiratory tube was folded.
Brusatol 2.7 ppm	Blackened larval bodies, folds in respiratory tubes, damage to the anterior part of the digestive tract and cuticle.
Brusatol 0.9 ppm	The digestive tract was damaged, the head was blackened. Respiratory tube folded. There was no damage to the cuticle.
Brusatol 0.3 ppm	Many folds on the respiratory tube, damage to the siphon, the head was blackened, no cuticle damage.
Brusatol 0.1 ppm	The respiratory tract/tube was folded, narrowed and damaged on the inside of the siphon.
Temephos 1 ppm	The respiratory and digestive tracts are severe damage with some dark spot. The siphon and anal gills were damaged/detached and blackened.
Control	Body condition intact and good, complete and good digestive system.

Bodies of control larvae (negative control) did not show any damages. *Cx. quinquefasciatus* larvae treated with various concentrations of brusatol and 1 ppm temephos exhibited damaged heads, cuticle, digestive tracts, breathing tract/tubes, siphons, and setae feathers.

## Discussion

### Larvicidal activity of brusatol against Cx. quinquefasciatus

The *Cx. quinquefasciatus* larvae used in this study were of instar 3 up to the beginning of instar 4. Larvae of instar 3 up to the beginning of instar 4 already have perfectly shaped organs, so death after treatment could not be attributed to the organs not being fully formed (14). The death of *Cx. quinquefasciatus* larvae was not caused by these environmental factors. The levels of pH, temperature, and room humidity in the media were still within the optimal pH range (6.5–7), temperature (25–27°C), and air humidity (60%–80%) for the development of *Cx. quinquefasciatus* larvae in bioassay research (11).

The Pesticide Commission (15) states that a larvicide is said to be effective if the death/dying number of larvae reaches at least 90% within 24 hours. Based on 24 hours observation results showed that in the control group (without treatment) there was no death of *Cx. quinquefasciatus* larvae, whereas at the lowest concentration of brusatol (0.1 ppm) there were 64% mortality of the larvae.

From Table 1, it shows that the higher the concentration of brusatol the higher percentage of death of *Cx. quinquefasciatus* larvae and statistically significant. Mortality of *Cx. quinquefasciatus* larvae 100% was found at the highest concentration of brusatol that was 24.3 ppm. This result is similar to previous research on *Ae. aegypti* larvae which proves that at the highest concentration of brusatol, it causes death of 100% larvae. At the lowest concentration of brusatol, it causes the death of *Ae. aegypti* larvae by 40% (8).

The difference in mortality of *Cx. quinquefasciatus* larvae may be influenced by several factors: larvae instar and different sensitivity of each test larva species. The larval instar factor is related to the age of the test larvae. Although the larvae used are larvae instar 3 up to the beginning of instar 4, but there are still variation in larval age. Differences in larval mortality are also influenced by differences in sensitivity of each test larva species. Although using larvae with the same instar and age, each larva has different levels of susceptibility. The interaction of toxic substances in the biological system is determined by the concentration of the test compound and the length of the test period (16).

Deaths of *Cx. quinquefasciatus* larvae of more than 90% were achieved at concentrations of brusatol 8.1 ppm and 24.3 ppm. This proves that brusatol is effective as a natural larvacide in *Cx. quinquefasciatus* larvae (15). Temephos at 1 ppm concentration caused 100% mortality of *Cx. quinquefasciatus* larvae, as well as at concentrations of 24.3 ppm brusatol. The mortality of *Cx. quinquefasciatus* larvae in this study was due to the toxic activity of the brusatol compounds acted as toxicants.

Brusatol toxicity on *Cx. quinquefasciatus* larvae is expressed by Lethal Concentration 50 and 90 (LC_50_, LC_90_), respectively 0.010 ± 0.122 and 0.654 ± 0.081 ppm. Sutiningsih & Nurjazuli (8), mentioned that brusatol toxicity (LC_50_, LC_90_) in *Ae. aegypti* larvae respectively of 0.669 ± 0.106 and 8.331 ± 0.060. The smaller the Lethal Concentration value the more toxic a compound is (17). This means that brusatol is more toxic to *Cx. quinquefasciatus* larvae than to *Ae. aegypti* larvae. The death of the mosquito larvae is caused by the inability of the larvae to detoxify the toxic compound that enters the body. The toxic compounds entering the larvae can cause four stages of response: excitation, convulsions, lysis (paralysis) and death (18). These toxic compounds cause disruption of the digestive system, respiratory system and nervous system in larvae (19–22). Brusatol enter the body of larvae through the mouth when the larvae were feeding. Brusatol is thought to decrease the activity of protease enzymes and food absorption and inhibit the taste receptors in the mouth area that will cause the larvae to fail to get a flavor stimulus, consequently the larvae are unable to recognize the surrounding food (23). Low feeding activity in the larvae causes the energy for the development of the larvae to be reduced so that the growth process is inhibited and eventually the larvae die. The bitter end of brusatol also causes food inhibition (antifeedant) in the test larvae. The bitter taste causes the larvae to not eat so the larvae starved and eventually die. Toxic compounds consumed by larvae will affect the amount and rate of eating so that resulted in decreased growth rate and survival ability. The toxicity power comes from its toxic substances that contained in the biological compounds of Makassar fruits. The substance can be toxic through the absorption of the gastrointestinal tract or through the skin of the larvae (26). Brusatol isolated from *B. javanica* (L.) Merr. has insecticidal and antifeedant properties against instar 3 larvae of *Spodoptera exigua* (7). The active material contained in *B. javanica* (L.) Merr. extract has larvicidal effect against *Crocidolomia pavonana* (Lepidoptera: Crambidae) (24, 25). Extract of *B. javanica* (L.) Merr. can inhibit feeding, decrease the rate of growth and inhibit laying *C. pavonana* imago (25). Bruceine A isolated from the seeds of *B. javanica* (L.) Merr can inhibit the growth and development of *Ae. aegypti* larvae to become pupa/adult (9).

### Morphological changes of Cx. quinquefasciatus

Observation of morphological changes is intended to determine the target of damaged organs/body parts of *Cx. quinquefasciatus* larvae after treatment with brusatol. The morphological observation of *Cx. quinquefasciatus* larvae after brusatol treatment showed differences compared with control (without treatment). From Fig. 1. it is seen that control of *Cx. quinquefasciatus* larvae (without treatment) do not show any damage to their body parts. The condition of the larvae is intact and good. Digestive and respiratory tract complete and well.

Observation of morphology of *Cx. quinquefasciatus* larvae after the treatment with lowest concentration of brusatol (0.1 ppm) showed that the respiratory tube was folded, narrowed and damaged on the inside of the siphon. Anal gills were partially detached and damaged (Fig. 2B). At higher concentrations of brusatol (0.9 ppm) showed more severe damage to the respiratory tract with more folds and damaged at the end of the tube leading to siphon (Fig. 2A). The body of the larvae and the head were blackened, the digestive tract was damaged while the cuticle was not. The higher the concentration of brusatol, the morphological damage of *Cx. quinquefasciatus* larvae was getting worse and widespread to cause damage to the digestive tract and cuticle. In addition, respiratory tubes, siphon and anal gills were having more severe damage.

Based on the results of this study, it proves that toxic substances in brusatol cause morphological damage in the body of *Cx. quinquefasciatus* larvae, such as head, cuticle, digestive tract, respiratory tract, siphon and anal gills. Brusatol is thought to enter the body of the larvae through the pores of the skin, the digestive tract / mouth and the respiratory tract/siphon (8). Toxic substances are relatively easier to penetrate the cuticle and extend into the body of the larvae (7). The number of toxic compounds that enter causes damage to skin cells. This toxic compound hydrolyzes the skin membrane by breaking down the skin protein (collagen) into several parts. The destruction of skin cell membranes resulted in the loss of impermeable membrane of the skin, so other toxic compounds are free to enter the body of the larvae (19). The large number of toxic compounds that enter causes the proteins in the skin membrane to be damaged, so the function of the skin as a body protector is disrupted (26).

Another way to insert brusatol into the body of *Cx. quinquefasciatus* larvae is through the respiratory tract. Air enters through a siphon attached to the water surface (16). Brusatol toxic compounds is likely covering the surface of the medium thus blocking the siphon from obtaining oxygen from the surface of the medium (26, 27). Neural tissues of the larvae are very sensitive to the lack of oxygen causing the wither on the nerves and damage on the siphon and as a result the larvae have difficulty in breathing and eventually die (28).

Brusatol toxic compounds also cause electrolyte imbalance in the anal region causing larvae strongly bite anal gills and resulted in severe damage to the anal gills (Fig. 2B). This result is supported by previous studies on *Ae. aegypti* larvae after treatment with 9 ppm of brusatol showed the presence of neurotoxic symptoms characterized by the aggressive circular motion of larvae biting the anal gills (8). Similar observations have also been reported by Warikoo and Kumar (29) on *Ae. aegypti* (L) larvae with *Argemone mexicana* root extract treatment concentration of 91.331 ppm and 156.684 ppm for 24 hours, showed a structure deformity on anal gills. There was a structural deviation/damage to anal papillary of early instar 4 of *Ae. aegypti* larvae after treatment with stem and leaf extract of *Achyranthes aspera* (12). The damages on anal papillae with shrinking cuticle has also been observed by Insun et al. (30) on the larvae *Cx. quinquefasciatus* after treatment with ethanol extract of *Kaempferia galanga*. Anal gills damage causes impaired function in osmotic and ionic regulation (20). This disorder of regulatory function is expected to be the cause of death of larvae *Cx. quinquefasciatus*. Brusatol can induce apoptosis in the insect cell line IOZCAS- Spec -II and Sf21 characterized by DNA fragmentation, caspase-3 activation and release of cytochrome-c enzyme from mitochondria (7).

Damage to the gastrointestinal tract and siphon in *Cx. quinquefasciatus* larvae began to be seen in treatment with brusatol at the lowest concentration (0.1 ppm). Digestive tract of *Cx. quinquefasciatus* larvae began to show the damage after brusatol treatment concentration of 0.9 ppm (Table 2). The results of this study were not different from previous studies, proved that bruceine A at 0.2 ppm concentration causes damage to the digestive and respiratory tracts of *Ae. aegypti* larvae (8). Distortion in the middle of the gastrointestinal tract (midgut) with a loss of pigmentation and a partial or total damage to cells in the larvae *of Ae. aegypti* after treatment with stem and leaves extract of *A. aspera* (12).

Microscopic observation by using a light/electron microscope showing at observations of 6,12, 24 and 48 hours, there was an increase of damage in epithelial midgut after treatment with *A. aspera.* Similar observations in midgut were also reported by Chaithong et al. (21) in *Ae. aegypti* larvae after treatment with pepper extract. Damage to the gastrointestinal tract and respiratory caused the metabolism and respiration of the larvae were disturbed so that the larvae became dead.

The results of this research suggest that brusatol can be categorized as abdominal and respiratorical toxic because it kills *Cx. quinquefasciatus* larvae with action target on gastrointestinal and respiratory tract. These results also support the potential of natural, plant-based larvicides for environmentally sensitive control of disease vectors such as *Cx. quinquefasciatus*.

This study will help the researchers to discover the critical areas of target site of brusatol, which has not been explored yet. Future studies should examine how brusatol affects growth hormone production as well as molting and metamorphosis also detailed description of histological damage by microscopic observation using transmission electron microscopy and fluorescence imaging of cell viability markers via confocal microscopy.

## Conclusion

Brusatol isolated from *B. javanica* (L.) Merr has a natural larvacidal activity in *Cx. quinquefasciatus* larvae that can be used to control the population of *Cx. quinquefasciatus* larvae as disease vectors. The larvicidal activity of brusatol is indicated by morphological damage to the body parts of *Cx. quinquefasciatus* larvae, such as in: head, cuticle, respiratory tract, siphon, digestive tract and anal gills.

## Ethical considerations

Ethical issues (Including plagiarism, informed consent, misconduct, data fabrication and/or falsification, double publication and/or submission, redundancy, etc.) have been completely observed by the authors.
